# When Is Improvement Possible? A Necessity‐Based Analysis of Short‐Term Eating Disorder Symptom Improvement During Inpatient Treatment

**DOI:** 10.1002/eat.70131

**Published:** 2026-05-11

**Authors:** Paolo Meneguzzo, Patrizia Todisco

**Affiliations:** ^1^ Department of Neuroscience University of Padova Padova Italy; ^2^ Padova Neuroscience Center University of Padova Padova Italy; ^3^ Eating Disorder Unit, Casa di Cura “Villa Margherita” – KOS Group Arcugnano Italy; ^4^ Psyco‐Nutritional Center Verona, Vicenza Italy

**Keywords:** eating disorders, EDE‐Q improvement, functional impairment, inpatient treatment, necessary condition analysis, treatment outcome

## Abstract

**Objective:**

Eating‐disorder–specific functional impairment may represent a boundary condition for treatment response, yet this possibility has rarely been examined. We investigated whether baseline functional impairment constrains short‐term eating‐disorder symptom improvement, indexed by change in EDE‐Q Global scores during inpatient treatment using a necessity‐based framework.

**Method:**

We retrospectively analyzed 462 inpatients who completed the Eating Disorder Examination Questionnaire (EDE‐Q 6.0) at admission and discharge. Functional impairment (Clinical Impairment Assessment, CIA), general psychopathology (SCL‐90‐R GSI), and body uneasiness (BUT‐GSI) were assessed at baseline. Short‐term eating‐disorder symptom improvement was defined as clinically meaningful improvement based on the Reliable Change Index and as continuous change in EDE‐Q Global scores. Necessary condition analysis with permutation testing and bottleneck analysis was applied, with subgroup analyses for anorexia nervosa versus non‐anorexia nervosa.

**Results:**

ED‐specific functional impairment emerged as a statistically significant necessary condition for clinically meaningful improvement (NCA *d* = 0.167, *p* = 0.011) and symptom change (*d* = 0.159, *p* = 0.002). Neither general psychopathology nor body uneasiness met criteria for necessity. Bottleneck analysis showed that larger symptom reductions occurred only above increasing CIA thresholds. Necessity effects were robust in AN but absent in non‐ diagnoses.

**Discussion:**

Functional impairment appears to constrain short‐term inpatient EDE‐Q improvement in anorexia nervosa, whereas no necessity effect was observed in non‐anorexia nervosa diagnoses. These findings suggest that ED‐specific functional impairment may function as a boundary condition for short‐term EDE‐Q improvement in anorexia nervosa within the present sample, whereas no corresponding necessity effect was observed for general psychopathology, body uneasiness, or non‐anorexia nervosa diagnoses.

## Summary

- Eating‐disorder–specific functional impairment at admission emerged as a necessary condition for short‐term symptom improvement during inpatient treatment, whereas general psychopathology and body image distress did not.
- Necessity‐based analysis suggested an observed boundary pattern in which clinically meaningful improvement was not observed in this sample at lower levels of functional impairment, highlighting boundary conditions for change rather than probabilistic predictors.
- This necessity effect was specific to anorexia nervosa, suggesting that ED‐specific functional impairment may be more closely related to short‐term EDE‐Q improvement in underweight presentations than in other diagnostic groups.
- In the present sample, a necessity‐based approach provided complementary information to conventional outcome thinking by highlighting a possible boundary pattern for short‐term EDE‐Q improvement.

## 1 Introduction

Eating disorders (EDs) are severe psychiatric conditions characterized by persistent disturbances in eating behaviors, body‐related cognitions, and psychosocial functioning (Attia and Walsh 2025). Although evidence‐based treatments are available, outcomes, particularly in inpatient settings, remain highly variable (Solmi et al. 2024). Identifying which baseline clinical dimensions are most relevant for treatment response therefore remains a central challenge in ED research (Chang et al. 2021; Meneguzzo et al. 2024).

Most outcome studies rely on association‐based approaches, such as regression models, to identify variables associated with symptom change on average (Kazdin 2007; Solmi et al. 2024). While informative, these approaches are less suited to examining whether some baseline characteristics may function as boundary conditions for change, that is, whether certain levels of improvement are not observed unless a predictor reaches a minimum range (Dul 2016).

ED‐specific functional impairment may be especially relevant in this context. Unlike symptom severity measures, functional impairment captures the extent to which ED symptoms interfere with social, occupational, and interpersonal functioning (Bohn et al. 2008). Previous research has linked functional impairment to illness burden, reduced quality of life, and treatment utilization in ED populations (Meneguzzo et al. 2020; van Hoeken and Hoek 2020). Body image disturbance and general psychopathology are also clinically relevant dimensions that have been linked to ED severity and outcome in prior research (Barakat et al. 2023; Fairburn et al. 2003; Jackson et al. 2022; Meneguzzo et al. 2025). Addressing this question may benefit from a necessity‐based framework, which can complement association‐based approaches by examining whether some baseline variables function as boundary conditions for change rather than as average predictors (Dul 2016; Marchetti, Koster, and Dul 2025).

Addressing this question may benefit from a necessity‐based framework. Necessary condition analysis (NCA) addresses a different question from conventional regression‐based models. Rather than estimating whether higher baseline values are associated with greater improvement on average, it examines whether some levels of the outcome are not observed when the predictor remains below a certain range. In this sense, NCA is best understood as a complementary descriptive method for exploring possible boundary conditions in the data, rather than as a replacement for more familiar association‐based approaches.

On this basis, the present study applies NCA to examine whether eating‐disorder–specific functional impairment, general psychopathology, and body uneasiness assessed at admission constitute necessary conditions for short‐term eating‐disorder symptom improvement during inpatient treatment, indexed by change in EDE‐Q Global scores. Given the distinctive clinical features of anorexia nervosa (AN), diagnostic subgroup analyses were conducted.

## 2 Methods

### 2.1 Study Design and Participants

This study is based on a retrospective observational cohort drawn from a specialized inpatient eating disorder program. Consecutive patients admitted to the Eating Disorder Unit of Casa di Cura Villa Margherita (Arcugnano, Italy) between June 2014 and December 2024 were considered for inclusion.

Inclusion criteria were (a) admission to the inpatient eating disorder program with a DSM‐5 eating disorder diagnosis, including A (restrictive or binge‐purge subtype), bulimia nervosa, binge‐eating disorder, avoidant/restrictive food intake disorder, atypical anorexia nervosa, and other specified feeding or eating disorders (OSFED); (b) age between 14 and 60 years; and (c) absence of severe psychiatric comorbidities likely to substantially interfere with assessment or treatment (e.g., schizophrenia spectrum or bipolar disorders), major neurological conditions, or severe medical illnesses. Common internalizing comorbidities, including depressive and anxiety disorders, were not exclusion criteria. Diagnoses were established by board‐certified psychiatrists with specific training in eating disorders and structured clinical interviewing, using structured clinical interviews based on DSM‐5 criteria (Shankman et al. 2018).

The present study is a retrospective analysis of data originally collected within an approved longitudinal observational study on inpatient treatment outcomes. All participants provided written informed consent for participation in that protocol and for the use of their clinical data for research purposes; for minors, consent was obtained from parents or legal guardians.

### 2.2 Measures

Eating disorder psychopathology was assessed using the Eating Disorder Examination Questionnaire 6.0 (EDE‐Q), with the Global score (range 0–6) indexing the overall severity of eating‐disorder–specific cognitions and behaviors over the preceding 28 days (Fairburn and Beglin 1994). Eating‐disorder–specific functional impairment was measured at admission using the Clinical Impairment Assessment (CIA) total score, which assesses the degree to which ED symptoms interfere with psychosocial functioning across personal, social, and cognitive domains, with higher scores indicating greater impairment (Welch et al. 2011).

General psychopathology was assessed using the Global Severity Index (GSI) of the Symptom Checklist‐90‐Revised (SCL‐90‐R), a widely used measure of overall psychological distress (Derogatis and Lazarus 1994). Body image–related distress was measured using the Global Severity Index of the Body Uneasiness Test (BUT‐GSI), which captures global body‐related dissatisfaction and distress (Cuzzolaro et al. 2006). All instruments have demonstrated good psychometric properties and are commonly used in clinical eating disorder research.

### 2.3 Inpatient Treatment Setting

Inpatient treatment was delivered within a specialized eating disorder unit using a structured, multidisciplinary rehabilitation program integrating medical monitoring, nutritional rehabilitation, and psychotherapeutic interventions (for details see Meneguzzo et al. 2024; Todisco et al. 2020). Treatment consisted of a structured multidisciplinary inpatient program based on cognitive–behavioral therapy with eating‐disorder–specific components, integrated medical monitoring, elements of third‐wave psychotherapy, and cognitive rehabilitation interventions, delivered within a closely supervised clinical setting.

### 2.4 Outcome Definition

The primary outcome was clinically meaningful improvement in ED symptoms, operationalized using the Reliable Change Index (RCI) applied to the EDE‐Q Global score. Patients with RCI ≤ −1.96 were classified as responders. This cutoff corresponds to the conventional threshold for statistically reliable change, reflecting a change exceeding the 95% confidence interval of measurement error. This dichotomous indicator was included to complement the continuous change score with a clinically interpretable marker of reliable short‐term improvement. A secondary outcome was continuous symptom change, defined as the difference between admission and discharge EDE‐Q Global scores.

### 2.5 Statistical Analysis

Treatment‐related change was evaluated using paired‐sample *t*‐tests and within‐subject effect sizes (Cohen's dz). NCA was conducted using the Ceiling Envelopment–Free Disposal Hull (CE‐FDH) technique with 10,000 permutation tests. NCA evaluates whether an empty upper‐left region is present in the predictor–outcome space, consistent with the possibility that lower values of a predictor are not observed together with higher values of the outcome. The resulting ceiling line and effect size should be interpreted descriptively and cautiously, as they may be sensitive to extreme observations, sparse regions of the predictor–outcome space, and sample‐specific variability. In line with methodological recommendations, a variable was considered a necessary condition only if the NCA effect size (*d*) was ≥ 0.10 and the permutation‐based *p* value was < 0.05. Bottleneck analyses were performed for variables meeting necessity criteria to estimate minimum levels required for specific degrees of symptom improvement. Diagnostic subgroup analyses were conducted for anorexia nervosa and non‐anorexia nervosa groups. As a sensitivity analysis, the RCI classification was recalculated using reliability coefficients ranging from 0.70 to 0.90. To assess robustness, we also performed a leave‐one‐out sensitivity analysis, in which the NCA model was iteratively re‐estimated after removing one observation at a time to evaluate whether the observed necessity effect was disproportionately influenced by individual cases.

## 3 Results

### 3.1 Sample Characteristics and Symptom Change

Patients included in the analyses (*n* = 462) did not differ from those without discharge data (*n* = 123) in age (27.8 ± 12.4 vs. 25.8 ± 11.5 years; *t* = 0.86, *p* = 0.39), sex distribution (female: 95.7% vs. 99.2%; *χ* ^2^ = 2.53, *p* = 0.11), or baseline EDE‐Q Global scores (3.82 ± 1.38 vs. 4.07 ± 1.39; *t* = −1.70, *p* = 0.09). Diagnostic composition was also comparable between groups (*χ* ^2^ = 6.84, *p* = 0.14): included patients comprised 37.7% AN restrictive subtype (AN‐R), 19.5% AN binge‐purge subtype (AN‐BP), 19.7% bulimia nervosa, 12.8% binge‐eating disorder, and 10.2% OSFED/other diagnoses, whereas patients without discharge data comprised 27.8% AN‐R, 28.7% AN‐BP, 21.7% bulimia nervosa, 10.4% binge‐eating disorder, and 11.3% OSFED/other diagnoses. These findings indicate no evidence of systematic attrition related to baseline clinical severity or diagnosis. See Table 1 for details.

**TABLE 1:** Sample characteristics and necessary condition analysis results.

Variable	Mean (SD) or %	NCA *d*	*p*
Sample characteristics			
Age (years)	27.3 (11.5)	—	—
Female sex (%)	96.4%	—	—
Anorexia nervosa (%)	56.4%	—	—
EDE‐Q Global (admission)	3.87 (1.39)	—	—
Variables tested as necessary conditions			
CIA total	32.7 (11.0)	0.167	0.011
SCL‐90‐R GSI	1.75 (0.69)	0.055	0.53
BUT‐GSI	2.89 (1.06)	> 0.001	1.00

*Note:* Values are reported as mean (SD) or percentage. ED psychopathology was assessed with the Eating Disorder Examination Questionnaire Global score (EDE‐Q); functional impairment with the Clinical Impairment Assessment (CIA); general psychopathology with the Symptom Checklist‐90‐Revised Global Severity Index (SCL‐90‐R GSI); and body uneasiness with the Body Uneasiness Test Global Severity Index (BUT‐GSI). Necessary condition analyses used the CE‐FDH method with 10,000 permutations. NCA effect sizes (*d*) and *p* values refer to clinically meaningful improvement (RCI ≤ −1.96). Dashes indicate variables not tested.

At admission, the mean EDE‐Q Global score was 3.87 (SD = 1.39), decreasing to 2.50 (SD = 1.37) at discharge. This reduction was statistically significant, *t*(461) = 21.90, *p* < 0.001, with a large within‐subject effect size (dz = 0.97). Based on the RCI, 127 patients (27.4%) met criteria for clinically meaningful improvement.

### 3.2 NCA

**FIGURE 1:**
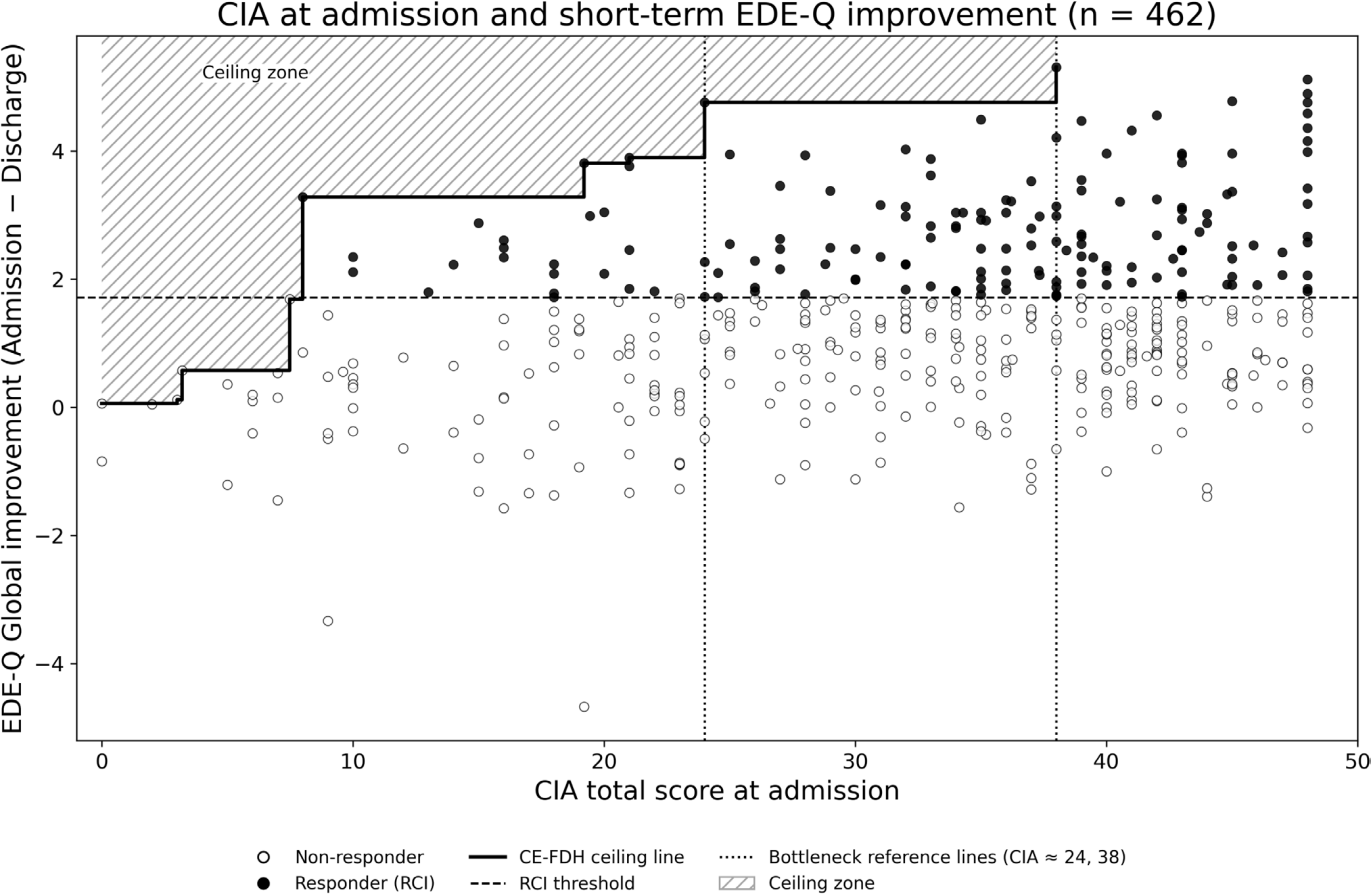
Necessary condition analysis (NCA) showing eating‐disorder–specific functional impairment at admission (CIA total score) as a boundary condition for short‐term symptom improvement in EDE‐Q Global scores (admission minus discharge). The hatched upper left region represents the ceiling zone, that is, the portion of the predictor–outcome space in which no observations were present. In NCA, this empty region indicates that higher levels of short‐term EDE‐Q improvement were not observed at lower CIA values in the present sample.

When clinically meaningful improvement was examined as a dichotomous outcome, only higher CIA total scores met the predefined necessity criteria (*d* = 0.167, *p* = 0.011), indicating a boundary pattern in the data whereby reliable improvement was not observed in this sample at lower levels of ED‐specific functional impairment. In contrast, neither general psychopathology (SCL‐90‐R GSI: *d* = 0.055, *p* = 0.53) nor body uneasiness (BUT‐GSI: *d* = 0.000, *p* = 1.00) met criteria for necessity. See Figure 1 for graphical representation.

Using continuous symptom change as the outcome yielded a comparable pattern. Again, only higher CIA scores met the predefined necessity criteria (*d* = 0.159, *p* = 0.002), whereas general psychopathology and body uneasiness did not.

### 3.3 Bottleneck Analysis

Bottleneck analysis was used to describe the CIA values at which larger EDE‐Q reductions were observed in the present sample. Results indicated a graded boundary pattern, whereby progressively larger reductions in EDE‐Q Global scores were observed only above increasing CIA levels. Specifically, reductions of approximately four points on the EDE‐Q Global scale were observed only among patients with CIA scores of at least 24 at admission, whereas reductions of five points or more were observed only among patients with CIA scores of approximately 38 or higher. Below these illustrative values, such levels of improvement were not observed in this dataset. These values should be interpreted cautiously as descriptive features of the present sample rather than as definitive clinical cut‐offs, particularly because larger EDE‐Q reductions are more likely to occur among patients with greater baseline symptom severity.

To address reliable improvement more directly, we examined the proportion of patients achieving reliable change across ranges of baseline functional impairment. In the present sample, among patients with CIA scores below 24, 31.0% (35/113) achieved reliable improvement. This proportion increased to 62.0% (111/179) among those with CIA scores between 24 and 38, and was 55.9% (95/170) among those with CIA scores above 38. These descriptive patterns suggest that lower CIA values were associated with a reduced proportion of patients meeting criteria for reliable improvement. These proportions are presented for descriptive purposes and do not imply a probabilistic model, but rather provide an intuitive illustration of the boundary pattern identified by the necessity‐based analysis.

### 3.4 Diagnostic Subgroup Analyses

In the anorexia nervosa group, functional impairment remained a significant necessary condition for improvement, with NCA effect sizes ranging from *d* = 0.18 to 0.20 across outcome definitions (all *p* < 0.05). No necessary conditions were identified in the non‐anorexia nervosa group. Sensitivity analyses varying the assumed reliability coefficient from 0.70 to 0.90 yielded a materially comparable pattern, with CIA remaining the only variable meeting the predefined necessity criterion across the tested range. Leave‐one‐out analyses showed that the CIA necessity effect remained stable across iterations and did not fall below the prespecified effect‐size threshold, supporting the robustness of the observed boundary pattern.

## 4 Discussion

This study applied a necessity‐based analytical framework to examine which baseline clinical dimensions constrain short‐term symptom improvement during inpatient treatment for eating disorders (Dul 2016; Marchetti, Colpizzi, et al. 2025). Eating‐disorder–specific functional impairment was the only variable that met the predefined necessity criteria for improvement, with this necessity effect observed in the anorexia nervosa group but not in the non‐anorexia nervosa group. Accordingly, the present findings should be interpreted as referring to short‐term change in ED psychopathology, rather than to global inpatient treatment success.

The distinction between necessity and sufficiency is central to interpreting these findings (Dul 2016; Marchetti, Koster, and Dul 2025). Functional impairment did not operate as a conventional probabilistic predictor of improvement; rather, lower CIA levels were associated with a boundary pattern in the data, with clinically meaningful change not observed in this sample at lower levels of ED‐specific functional impairment. This boundary‐condition logic extends traditional outcome research, which has largely focused on average predictors of change, by examining whether some baseline characteristics are observed when improvement occurs, rather than only whether they are associated with greater change on average (Dingemans et al. 2016; O'Brien et al. 2017; Vall and Wade 2015).

The diagnostic specificity of this necessity effect in is clinically and theoretically informative. Anorexia nervosa is characterized by pronounced egosyntonicity, cognitive rigidity, and ambivalence toward change, which may limit engagement with treatment when symptoms remain functionally tolerable (Gregertsen et al. 2017; Tenconi et al. 2021; Williams and Reid 2010). In this context, eating‐disorder behaviors may retain perceived adaptive or identity‐stabilizing functions until their impact on everyday functioning becomes sufficiently disruptive. Functional impairment may therefore mark a clinical context in which short‐term EDE‐Q improvement is more often observed, although the present design does not permit conclusions about the mechanisms underlying this pattern (Fairburn et al. 2003).

Importantly, these findings should not be interpreted as suggesting that greater impairment causes improvement. Rather, they indicate that below a certain level of functional interference, improvement may be structurally constrained, particularly in restrictive eating‐disorder presentations. This necessity‐based perspective helps explain why patients with comparable levels of symptom severity may show markedly different treatment responses and underscores the importance of distinguishing between symptom intensity and functional consequences in clinical assessment (Bohn et al. 2008; Mond et al. 2005).

The absence of a necessity effect for body image disturbance indicates that baseline BUT‐GSI scores did not function as a boundary condition for short‐term EDE‐Q improvement in the present dataset. This should not be interpreted as evidence that body image disturbance is unimportant to treatment response, but only that it did not meet necessity criteria within this specific sample and analytic framework. This interpretation remains compatible with previous evidence showing that body image–related processes may still be clinically relevant in intensive treatment, even if they do not emerge here as necessary conditions for short‐term symptom change (Eshkevari et al. 2014; Zipfel et al. 2014).

### 4.1 Clinical Implications

These findings suggest that assessing ED–specific functional impairment at admission may provide clinically relevant information beyond symptom severity alone. In inpatient settings, if replicated, these findings may suggest that patients with relatively low ED‐specific functional impairment at admission, particularly in AN, represent a subgroup in which short‐term EDE‐Q improvement is less frequently observed. This pattern may warrant closer attention to treatment engagement, although no intervention‐specific conclusions can be drawn from the present design. Moreover, the absence of necessity effects for body image disturbance supports a phased treatment approach, in which symptom stabilization and functional recovery may precede more intensive work on body‐related distress. Necessity‐based frameworks may thus complement traditional outcome models by informing individualized treatment planning.

### 4.2 Limitations and Implications

These findings should be interpreted within the limits of a retrospective observational design and a necessity‐based analytic framework, both of which support associative rather than causal inference. In addition, the estimated ceiling line and ceiling zone may be influenced by extreme observations, sparse regions of the predictor–outcome space, and sample‐specific variability. Accordingly, these features should not be interpreted as fixed population‐level thresholds and require replication in independent samples. This pattern may also partly reflect the mathematical constraint that larger reductions are more likely among patients with higher baseline EDE‐Q scores, which are themselves correlated with higher CIA levels. Moreover, results are restricted to short‐term inpatient outcomes and require replication across different levels of care to assess their generalizability. In addition, the present study focused specifically on short‐term change in EDE‐Q Global scores, which captures eating‐disorder psychopathology but does not fully reflect other key inpatient treatment targets such as weight restoration or behavioral symptom remission.

## 5 Conclusions

This study provides evidence that ED‐specific functional impairment delineates a boundary condition for short‐term inpatient EDE‐Q improvement in the anorexia nervosa group, whereas no corresponding necessity effect was observed in non‐anorexia nervosa diagnoses. In this sample, larger short‐term EDE‐Q improvements were observed primarily at higher levels of ED‐specific functional impairment, consistent with a boundary‐condition interpretation. In this context, a necessity‐based approach offered a complementary way of examining whether some baseline variables may function as boundary conditions for change, alongside more conventional association‐based models.

## Author Contributions

**Patrizia Todisco:** conceptualization, investigation, funding acquisition, methodology, validation, project administration, resources, supervision, writing – review and editing. **Paolo Meneguzzo:** conceptualization, investigation, writing – original draft, methodology, validation, visualization, software, formal analysis, data curation.

## Funding

The authors have nothing to report.

## Ethics Statement

The authors assert that all procedures contributing to this work comply with the ethical standards of the relevant national and institutional committees on human experimentation and with the Declaration of Helsinki of 1975, as revised in 2008. The research protocol obtained specific approval from the Vicenza Ethics Committee (47/21).

## Consent

Written informed consent was obtained from all participants, or parents or legal guardians if the participants were under the age of 18 years, for their participation in this study and inclusion in any published research article.

## Conflicts of Interest

The authors declare no conflicts of interest.

## Data Availability

The data that support the findings of this study are available on request from the corresponding author. The data are not publicly available due to privacy or ethical restrictions.
